# Noncommunicable Diseases, Park Prescriptions, and Urban Green Space Use Patterns in a Global South Context: The Case of Dhaka, Bangladesh

**DOI:** 10.3390/ijerph17113900

**Published:** 2020-05-31

**Authors:** S.M. Labib, Faysal Kabir Shuvo, Matthew H. E. M. Browning, Alessandro Rigolon

**Affiliations:** 1 School of Environment, Education and Development, University of Manchester, Oxford Road, Manchester M13 9 PL, UK; 2 School of Health and Society, Faculty of Social Sciences, University of Wollongong, Wollongong, NSW 2522, Australia; smfks513@uowmail.edu.au; 3 Department of Parks, Recreation, and Tourism Management, Clemson University, Clemson, SC 29631, USA; mhb2@clemson.edu; 4 Department of City and Metropolitan Planning, The University of Utah, Salt Lake City, UT 84112, USA; alessandro.rigolon@utah.edu

**Keywords:** urban green space, urban parks, park prescriptions, nature prescriptions, noncommunicable diseases, urban health, health promotion, Global South, developing countries

## Abstract

Urban green space use is often associated with improved physical and mental health and lower noncommunicable disease (NCDs) burdens. Factors that influence green space visits have been documented in cities of the Global North, but evidence of urban green space use patterns for cities in the Global South is scarce. The aim of this study is to investigate factors influencing urban green space use patterns in Dhaka, Bangladesh, a megacity of the Global South, with a particular focus on how poor health condition and healthcare professionals’ prescriptions to exercise outdoors (park prescriptions—ParkRx) impact the green space use of middle-aged adults. We collected green space characteristics and use factors (i.e., availability, accessibility, attractiveness, and attachment), health condition, ParkRx, and urban green space use intensity (i.e., frequency and duration) via a self-reported questionnaire from 169 middle-aged residents of Dhaka. We used multivariate modeling to estimate the association of green space characteristics, health condition, and ParkRx with use intensity. We further applied a mediation analysis to determine the influence of ParkRx on the relationship between residents’ poor health conditions and use intensity. We found that green space availability and accessibility did not significantly influence use intensity, but attractiveness was negatively associated with use intensity. Green space use intensity was significantly and positively associated with attachment to the green space, poor health condition (i.e., having noncommunicable diseases), and ParkRx. ParkRx significantly mediated the relationship between health condition and use intensity. We observed limited supply, poor access, and low attractiveness when studying the urban green spaces in Dhaka, but these qualities did not affect use intensity, as found in many case studies in the Global North. In contrast, urban green space use intensity in our case study is mostly dependent on poor health condition and park prescriptions.

## 1. Introduction

Urban green space—including parks, gardens, and other open spaces—can help promote positive health outcomes and prevent the risk of several chronic and non-communicable diseases [1,2,3,4,5]. Specifically, contact with urban green spaces can support human health through different pathways, promoting physical activity and social interactions, fostering psychological restoration, and reducing harm from hazardous environmental exposures, such as air pollution [6,7,8,9]. Notably, outdoor physical activity minimizes the adverse physiological effects stemming from sedentary lifestyles, such as the development of chronic health problems (e.g., type 2 diabetes) [10,11,12,13]. The intertwined effects of green spaces on increased physical activity and social interactions also boost cognitive and psychological benefits [6,14,15,16,17].

Most studies on the benefits of urban green spaces for human health have been conducted in Global North countries, which have higher incomes and levels of industrialization [6,18,19]. In contrast, significantly fewer studies on these benefits have focused on Global South countries, which are also known as developing countries [20,21,22,23]. The Global South includes low- and middle-income countries with lower rates of industrialization and encompasses all of Africa and South America and most countries in Asia [18,24]. Although the Global South includes very diverse countries (and diverse cities and rural areas within such countries), it has a few unique characteristics that make the green space–health relationship particularly germane to study. Many Global South countries include several megacities (more than 10 million residents), where rapid population growth has put increasing pressure on urban infrastructure and has led to significant population density [21,25,26]. Furthermore, the large population sizes of megacities stress the local supply of natural resources, including green spaces and waterbodies [27,28,29].

Researchers have identified several factors associated with green space use patterns and subsequent health benefits. These factors include but are not limited to the availability (e.g., amount), accessibility (e.g., distance), and attractiveness (e.g., perceived quality) of green spaces; sociodemographic characteristics of users; and knowledge of the health benefits of green space contact [30,31,32,33,34,35]. Most of these factors have been investigated in the context of Global North countries, but several researchers argued that factors influencing the green space use patterns in Global North countries might not have similar influences for populations in Global South countries [20,23,36,37]. In particular, the availability and accessibility of green spaces, sociodemographic and religious characteristics of users, and cultural contexts often vary between the Global North and South [21,24,36,38]. Given these differences, the findings on what predicts green space use patterns in Global North contexts might not be generalizable to cities in the Global South.

Among the factors associated with green space use patterns, scholars have increasingly focused on motivations stemming from knowledge of the health benefits of green space contact [33,39,40,41,42]. Furthermore, healthcare professionals are increasingly seeking to motivate people to visit green spaces, resulting in park prescription or green prescription programs (hereafter, ParkRx) [43]. Through these programs, healthcare professionals formally recommend that their patients visit natural settings regularly and engage in physical activity to help manage chronic and noncommunicable diseases (NCDs) [33,40,44,45,46]. ParkRx programs fall under a wider umbrella of nature-based health interventions, along with horticultural therapy and forest bathing. Health care professionals in New Zealand were among the first to formally implement ParkRx programs in the 1990s [39,44], and other Global North countries have recently followed suit [33,41,42,47]. General practitioner centers in New Zealand prescribed three months of 30 min per day of outdoor physical activity to reduce the risk of chronic diseases [39]. In the USA, physicians used ParkRx to improve self-reported resilience among children [41,48]. In the UK, nature prescriptions were formally adopted by general practitioners in Shetland, Scotland [49]. In Singapore, ParkRx interventions were successfully used to increase physical activity in parks and improve psychosocial quality of life among middle-aged adults [35].

Despite the increase in ParkRx programs in Global North contexts, the scientific literature on its efficacy and patient adherence remains unclear. In particular, the quantity and quality of programs available for study are inadequate, which complicates the implementation of evaluation studies [33,35,40,44]. The focus on Global North countries does not address contexts where such programs or approaches may be particularly important, such as megacities in the Global South. Furthermore, very little research has studied ParkRx among populations similar to those the Global South, such as low-income and non-White people. 

In this study, we provide new evidence by investigating the influence of green space characteristics, health condition (i.e., non-communicable diseases), and ParkRx on green space use patterns among middle-aged adults using a case study approach set in the context of a Global South megacity. Our population includes middle-aged adults in one Global South country, who are expected to experience the greatest increase in chronic disease of any generation [38,50,51]. Our study site is comprised of green spaces in the megacity of Dhaka, Bangladesh. This study has three objectives: First, to describe urban green space use patterns in middle-aged adults in Dhaka, a megacity of the Global South, which is a relatively understudied context for green space use patterns; second, to identify green space characteristics that predict urban green space use patterns among middle-aged residents in Dhaka; and third, to determine the influence of health condition and ParkRx on green space use patterns in Dhaka. Evidence generated from this study can inform the implementation of ParkRx programs for health promotion and green space management in Bangladeshi cities and, to some extent, other urban areas of the Global South.

## 2. Materials and Methods 

### 2.1. Case Study Area

**Figure 1:**
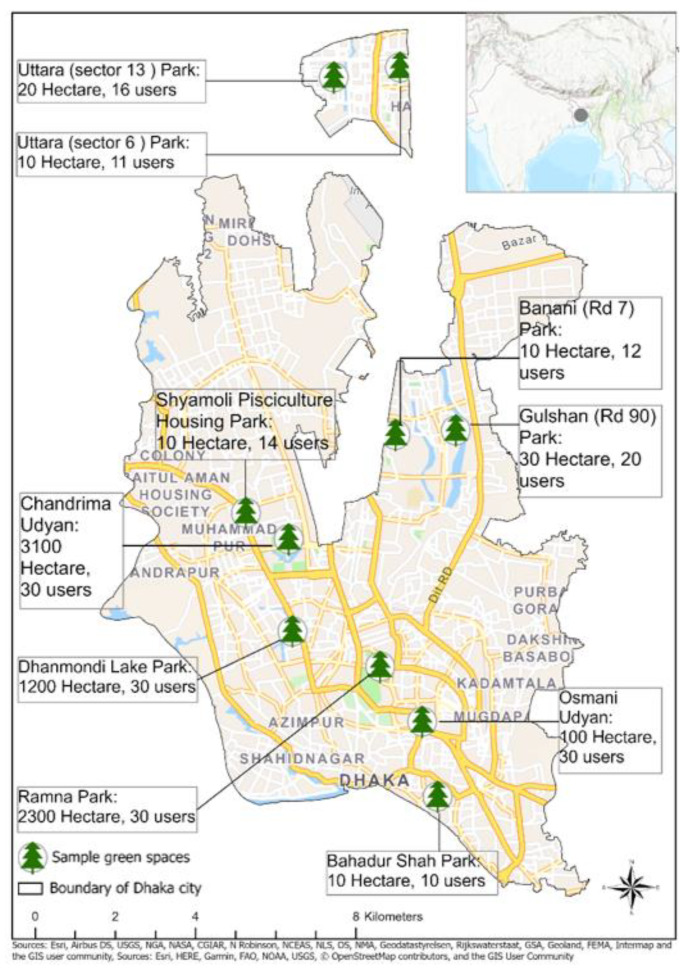
Study area location and studied green spaces in Dhaka.

We conducted this study in Dhaka, one of the fastest-growing megacities in the world. Dhaka, the capital city of Bangladesh, is located on the eastern banks of the Buriganga River (Figure 1). Dhaka is the leading financial, economic, and administrative hub of Bangladesh and generates a large share of the country’s gross domestic product. Estimates from 2015 suggest that over 17.6 million people inhabit the city’s 1528 km^2^ area. That population is expected to grow to nearly 26 million by 2035 [52]. For this study, we considered the spatial extent of the Dhaka City Corporation, which has a total area of approximately 134 km^2^ and an average population density of 1110 people/km^2^.

Dhaka’s rapid growth has resulted in significant urban sprawl and fast land use changes. Between 1975 and 2010, most of the city’s green infrastructure (e.g., open space, parks, water bodies) has been transformed into grey infrastructure (e.g., buildings, roads) [26,28,53]. Therefore, city residents have lost opportunities to connect with health-promoting natural landscapes. Furthermore, residents are increasingly exposed to traffic pollution that could have been absorbed by green spaces and vegetation land covers, were they preserved from development [54,55]. Dhaka is arguably one of the least livable cities in the world based on its decreasing amount of green space, increased air pollution, and traffic congestion [26,29,56,57,58]. 

**Figure 2:**
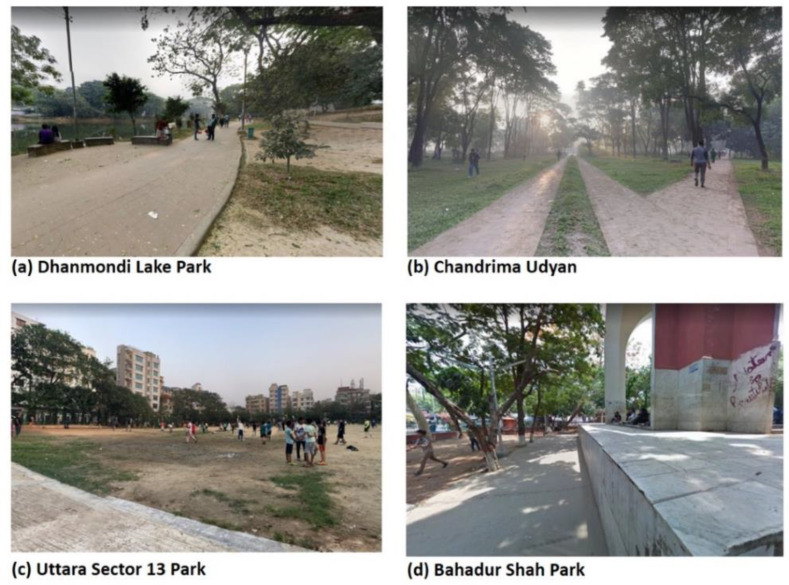
Examples of the conditions of urban green spaces in Dhaka City, Bangladesh (**a**–**d**). Images were collected from Google Street View.

Dhaka has only 54 green spaces, which include public parks and open spaces. These green spaces amount to 283.49 hectares—a small area given its large population (i.e., 0.039 hectares per 1000 residents) [57,59]. By comparison, New York City has more than 5000 parks covering more than 12,140 hectares, with approximately 1.4 hectares of green space per 1000 residents [60]. Perhaps as a result of the low acreage, most Dhaka residents do not have a park within a 20 min walk [57]. In contrast, standards set by Natural England indicate that residents should have access to green space within a 5 min walk for health promotion [61], and, in the USA, the Trust for Public Land calls for green space access within a 10 min walk of every resident [62]. This discrepancy between what is and what should be clearly illustrates the limited supply of accessible green spaces in Dhaka. Tabassum and Sharmin [63] also found that the majority of green spaces in Dhaka are not properly maintained, citing problems with littering, broken furniture, degraded trails, poor landscaping, and graffiti (Figure 2). 

### 2.2. Sample and Data Collection

We recruited 202 middle-aged green space users in 10 Dhaka green spaces using a convenience sample and interviewed them on-site with a structured questionnaire (available at https://github.com/labiblm/UGSusagDhaka). The questionnaire had four sections: Introduction, geographic/demographic characteristics, socioeconomic characteristics, and green space/health information. We developed this questionnaire based on questions from valid and reliable survey instruments, such as those of Shanahan et al. [64] and Schipperijn et al. [34], and we further confirmed the choice of questions through consultations with local researchers in architecture and urban planning.

For the data collection processes, we recruited and trained three research assistants. Training included classifying images of green space users across the age spectrum and reaching consensus on who would be considered middle-aged. People aged 45–65 years were the target [65,66].

#### Data Collection Sites

We defined urban green spaces as publicly accessible open spaces within the city that included considerable amounts of vegetation and that supported recreational activities, such as walking or playing sports [67,68]. Specifically, urban green spaces included parks, playgrounds, and botanical gardens, among other managed/unmanaged open spaces in Dhaka city. We selected the 10 green spaces through a stratified sampling procedure using wards, which are the smallest administrative units in the city. We stratified wards from the two city corporation regions (Dhaka North and Dhaka South) according to a composite socioeconomic index developed through Dhaka City census data, the Socio-Economic Profile for Disadvantaged (SEPD) [69]. We combined and weighted socioeconomic census variables on education, housing, and employment status to generate a cumulative score for each of the 92 wards. We then classified the wards into quartiles from ‘most disadvantaged’ to ‘least disadvantaged’. Finally, we randomly selected two green spaces from each quartile. 

In addition to the wards identified by the SEPD quartiles, we intentionally selected two large green spaces that were of high quality and were well maintained (i.e., Chandrima Udyan and Ramna Park) as data collection sites. This enabled us to include green spaces with significant variation in size and quality. Chandrima Udyan and Ramna Park are considered the most accessible parks in Dhaka [57,59,70]. Figure 1 shows the location of the green spaces included in the study, along with the sizes of the green spaces and the number of users surveyed. 

We conducted all field interviews in June 2017. Our survey targeted regular users of green spaces in Dhaka. Our questionnaire included questions on how long respondents were visiting the green space and how often they visited weekly. We trained the research assistants to discuss our intent to collect data from regular users with potential respondents before green space users participated. This minimized the number of respondents who were not regular users. To ensure the anonymity of respondents, we did not collect personal information (e.g., name, age, address). Each participant provided written consent. 

### 2.3. Measures

#### 2.3.1. Green Space Use Patterns

Green space use is the main outcome variable of this study. We used two items from past research to measure this concept: Frequency of visits and the average length of visits [34,64]. Participants reported how many urban green space visits they made per week on a four-point scale (1 = 1/none, 2 = 2–3, 3 = 4–5, 4 = more than 5). Furthermore, respondents provided the average duration of visits on another four-point scale (1 = less than 10 min, 2 = 10–20 min, 3 = 20–30 min, 4 = more than 30 min). We recoded these scores into binary variables to represent high versus low frequency/duration. Respondents visiting more than four days per week were considered high-frequency users (‘*frequency*’ = 1), whereas other respondents were coded as low-frequency users (‘*frequency*’ = 0). Respondents using the green space for more than 30 min per visit on average were considered long-duration users (‘*duration’* = 1). Other respondents were considered short-duration users (‘*duration*’ = 0). These thresholds were based on empirical guidelines suggesting that 30 min of brisk walking regularly, for a total of 150 min per week, can help a person achieve the majority of the protective effect of physical activity against chronic disease [71,72,73]. In addition to these guidelines, we observed that the distribution of the response variable was skewed (e.g., very low responses for less than 10 min visits). Thus, recoding provided an improved classification of use intensity over the initial variable. By combining these binary variables, we created a new outcome variable to obtain a comprehensive measure of green space use encompassing duration and frequency. A user who makes both high-frequency visits and stays for a long duration was considered a high-intensity user (‘*Use intensity*’ = 1). All other users were coded as low-intensity users (‘*Use intensity*’ = 0) (Table 1). 

#### 2.3.2. Green Space Characteristics 

We measured green space attractiveness and accessibility, two key factors that were likely to influence the use of green spaces among middle-aged residents of Dhaka. We considered green space attractiveness, which describes the perceived aesthetic qualities of a green space [68,74], because several studies have indicated that attractiveness is an important motivating factor for visiting green spaces and engaging in physical activity [75,76,77,78]. In our questionnaire, we asked the respondents to list and evaluate all of the attractive elements in the green space. We expected those elements to include vegetation, walkways, furniture (e.g., benches), fitness facilities, landscape design (e.g., lawns and fountains), water features (e.g., ponds), and playground equipment [30,31,68,77]. The respondents were asked to report the number of attractive features they found in the green space, and we recoded the attractiveness of the green space using a five-point Likert scale ranging from 1 = at least one attractive feature to 5 = more than five attractive features.

Green space accessibility is considered to be one of the most important factors related to green space use, and it is one of the most studied factors related to park usage and health outcomes [19,20,79]. Two types of accessibility have been previously discussed: Perceived proximity (e.g., travel time to walk to the green space) and objectively measured proximity (e.g., actual distance between a person’s home and the closest green space) [79,80,81]. Here, we chose perceived proximity as the measure of accessibility because previous studies showed that this measure is often a stronger motivator for green space use than objective proximity [82,83]. In our survey, we used an item from the Neighborhood Environment Walkability Scale (NEWS) to measure perceived accessibility [34,84]. We asked respondents to categorize the time it may take to walk (perceived walking time) to the green space they were visiting on a five-point scale (1 = more than 20 min, 2 = 16–20 min, 3 = 11–15 min, 4 = 5–10 min, and 5 = less than 5 min). We recoded these values into reverse order to ensure that larger numbers indicated greater perceived accessibility.

#### 2.3.3. Green Space Attachment

We considered user attachment to green space as an important factor for green space use. People can become attached to leisure spaces based on their community context, personal characteristics, and natural environmental context [85,86,87,88]. Clark et al. [87] and Lewicka [89] argued that the length of residence and local social capital are associated with place attachment. Several studies also indicated that place attachment and prior experiences in parks could motivate repeated visits, including frequency and type of use [85,88,90,91]. To measure attachment to green space, we asked respondents the number of years they had visited the green space they were in when they were interviewed. We recorded their responses on a five-point Likert scale (1 = less than or equal to 1 year, 2 = 2–3 years, 3 = 3–4 years, 4 = 4–5 years, and 5 = more than 5 years).

#### 2.3.4. Green Space Availability

We measured the local green space supply to quantify the availability of green spaces. Several studies indicated that at the residential level, green space supply is correlated with increased visitation and physical activity [36,37,92,93]. Here, we estimated supply by summing the total land area of green space polygons within or intersected by the administrative ward boundaries for each participant’s home. We then divided the total green space area by the number of people living in that ward to compute a normalized per-capita green space supply for each respondent.

#### 2.3.5. Health Condition and ParkRx

We selected the presence or absence of any non-communicable diseases (NCDs) as the primary health indicator of participants. According to the World Health Organization (WHO), in Bangladesh, there are approximately 580,000 deaths every year from NCDs, representing 67% of the total number of deaths in the country [94]. The WHO and the Bangladeshi government have already recognized NCDs as the most significant public health issue for Bangladeshi citizens. Previous studies reported that the most prevalent NCDs in Bangladesh are diabetes, cardiovascular disease, hypertension, chronic respiratory diseases, and cancer [95,96,97]. Based on these studies, we asked respondents about whether they reported having one of these NCDs. We recoded responses into a binary variable (1 = have a NCD/s, 0 = do not have a NCD/s). 

In Bangladesh, it is a common practice to prescribe outdoor physical activity as part of the 3D’s approach—Discipline, Diet, and Drugs—for treating patients with NCDs [98]. Considering such and based on previous studies, we included ParkRx as a potential motivating factor for green space visitation [33,35,46,99,100]. To identify ParkRx, we asked participants about the key reasons for visiting the green space where they were interviewed. We recorded their answer as a binary response regarding whether visitation related to a ParkRx (1) or any other reason/s (0). Specifically, we coded ParkRx as “1” if a healthcare professional had prescribed and recommended visiting a green space. 

#### 2.3.6. Confounders

We collected data on several potentially confounding variables in the questionnaire based on prior knowledge from studies showing the determinants of green space use patterns [19,101]. Variables included sex, level of educational achievement (i.e., none, secondary school, higher secondary school, bachelors, post-graduate), employment status (i.e., unemployed, retired, employed), and mode of travel to green spaces (e.g., walking, public transport, private vehicles). Because we targeted a specific age group, we did not collect information about respondents’ age, as we considered that the respondents would belong to the same age group (e.g., middle-aged).

### 2.4. Analyses

We first explored the descriptive statistics of the study population and cross-tabulation among variables such as NDCs, ParkRx, and use intensity. Then, we fitted logistic regression models to study whether green space characteristics, health condition, and ParkRx were associated with green space ‘*Use Intensity*’ while controlling for the aforementioned confounders. We also conducted a mediation analysis to investigate whether ParkRx mediates the relationship between health condition (i.e., NCDs) and ‘*Use Intensity*’. All analyses were performed with R (version 3.6.2; R Development Core Team, Vienna, Austria). Packages included MASS, car, and mediation [102,103,104]. The full dataset and R-markdown code can be found at https://github.com/labiblm/UGSusagDhaka.

#### 2.4.1. Main Analyses

We performed several multivariate logistic regression analyses to identify the associations between green space characteristics (attractiveness, accessibility, attachment, and green space per-capita), health condition (NCDs/Chronic), ParkRx, and ‘*Use intensity*’ (dependent variable). Model 1 included green space attractiveness, accessibility, attachment, and per-capita green space supply as independent variables. Model 2 included health condition (i.e., NCDs) as an independent variable in addition to the Model 1 variables. Model 3 included the independent variables from Model 1 and ParkRx as an additional independent variable. We adjusted all of the models by adding the confounders discussed in Section 2.3.6. We considered a *p*-value of 0.05 as an indicator of critical statistical significance and a *p*-value of 0.10 as an indicator of marginal significance. We used Akaike Information Criterion (AIC) values to compare the models’ fits, and we reported Nagelkerke’s pseudo-R^2^ values as an estimate of the models’ explanatory power. 

#### 2.4.2. Mediation Analyses

To analyze the direct and indirect effects of health condition (i.e., NCDs) and ParkRx on green space use intensity, we performed mediation analyses [105,106]. We hypothesized that it is likely that respondents with poor health condition (i.e., NCDs) often receive advice from healthcare professionals to perform indoor and outdoor physical activity. Thus, we investigated whether a recommendation of ParkRx (i.e., exercising outdoors in parks) is a possible influencing factor that mediates the direct association between poor health condition and use intensity. In this regard, we formulated a hypothesis (H1). We specified ParkRx as the mediator in the NCDs–use intensity association and assumed a causal pathway as follows: NCDs -> ParkRx -> green space use intensity.

**Hypothesis 1** **(H1).** *ParkRx significantly mediates the relationship between NDCs and green space use intensity*.

To test this hypothesis, we utilized a mediation package to estimate the direct, indirect, and total effects as well as the proportion mediated [104,106,107]. First, we specified a mediator model (ParkRx = NCDs + Green space characteristics + Covariates, Model 4), and an outcome model (Use intensity = NCDs + ParkRx + Green space characteristics + Covariates, Model 5). Next, we included the model objects (mediator model: Model 4 and outcome model: Model 5) in the mediation function and specified the independent variable (‘treat’ in the software package; here, this is NCDs) and ‘mediator’ (ParkRx) variable. In this case, we used binary terms for the independent (NCDs = 0 or 1) and mediator (ParkRx = 0 or 1) variables. Within the mediation operation, the function split the binary independent variable into two sample groups: ‘Control’ (i.e., without NCDs) and ‘treatment’ (i.e., with NCDs). For both groups, the mediation function estimated the average direct effect (ADE), average causal mediation effect (ACME), and proportion mediated for ParkRx. In this process, the average of both groups indicated overall effect sizes. We assessed the confidence intervals of all the effect sizes and proportions using 1000 nonparametric bootstrap simulations (see Tingley et al. [104]).

## 3. Results

### 3.1. Sample Characteristics

Of the 202 middle-aged green space users invited to participate, 169 completed the questionnaire (response rate = 83.7%). The majority were male (61.5%). A relatively high proportion (30%) had no formal education, and few had a bachelor’s degree (6.5%) (Table 2). Regarding occupation, 42% reported that they were unemployed or retired. Approximately 80% reported walking to the green space where they were surveyed, less than 10% reported traveling by public bus—public bus service is not satisfactory in Dhaka [108,109]—and less than 4% reported traveling by private car. Nearly three-quarters of respondents reported at least one chronic disease or illness (Table 2).

### 3.2. Green Space Characteristics and Use Patterns

Most respondents lived more than a 10 min walk from the green space they visited, and less than 10% lived within a 5 min walk (Table 3). Nearly two-thirds of respondents perceived the green space’s attractiveness as moderate or low. In terms of area-level green space supply, the mean green space per capita value was 2.29 m^2^/person (SD = 4.55). Almost half of the respondents visited the same green space for only one year, whereas fewer than 10% visited the same green space for more than five years. Nearly three-quarters used green spaces more than five days a week, but 70% spent less than 30 min per visit, on average. Therefore, approximately two-thirds of the respondents were classified as low-intensity users (Table 3). 

A cross-tabulation analysis elucidated associations amongst health condition, ParkRx, and ‘Use intensity’ groups (Table 4). As expected, most respondents without NCDs did not receive a ParkRx (96.0%), but nearly three-quarters of respondents with NDCs did receive a ParkRx (72.3%). These percentages suggest that receiving a ParkRx was highly dependent on the health condition of the respondents. Also, respondents without NDCs were mostly low-intensity users (80%), and a large share of respondents with NDCs were high-intensity users (40%) (Table 4). Among high-intensity users, the majority had NCDs (83%). These descriptive statistics suggest that high-intensity users mostly had NCDs, and people without NCDs were usually low-intensity users.

### 3.3. Predictors of Green Space Use Patterns

The logistic regression models we used to identify predictors of green space use intensity are presented in Table 5. Model 1 shows that attractiveness was significantly and negatively associated with use intensity. We also observed that accessibility did not have a significant association with use intensity. In contrast to attractiveness and accessibility, attachment to green space was significantly and positively associated with use intensity. A one-year increase in attachment corresponded to 2.10 times greater odds of the respondent being a high-intensity user. Finally, neighborhood-level green space per capita had no significant association with use intensity (Table 5).

#### 3.3.1. Influence of Health Condition and ParkRx on Green Space ‘Use Intensity’

Model 2, where we added NCD, shows similar results to those of Model 1 in terms of green space characteristics and use factors, but it exhibits greater explanatory power and lower prediction errors than Model 1 (increased R^2^ and lower AIC). In Model 2, poor health condition is a stronger significant predictor of use intensity than green space characteristics and use factors. Respondents with NCDs were 2.82 times more likely to engage in high-intensity green space use than those without NCDs (Table 5).

Model 3 shows a significant positive association between ParkRx and ‘Use intensity.’ Additionally, ParkRx was a stronger predictor of green space ‘Use intensity’ than green space characteristics. Respondents with a ParkRx were 6.32 times more likely to be high-intensity users (Table 5). Model 3 has higher explanatory power and lower prediction errors than Models 1 and 2. Attractiveness and attachment were significantly associated with use intensity in all three models. In contrast, both accessibility and green space per capita indicated non-significant associations with green space ‘Use intensity.’ Finally, both poor health condition and having a ParkRx yielded significant positive associations with use intensity. Including either of these two variables increased the model’s explanatory power and reduced model prediction errors.

#### 3.3.2. Mediation Effect of ParkRx on Green Space ‘Use Intensity’

**Figure 3:**
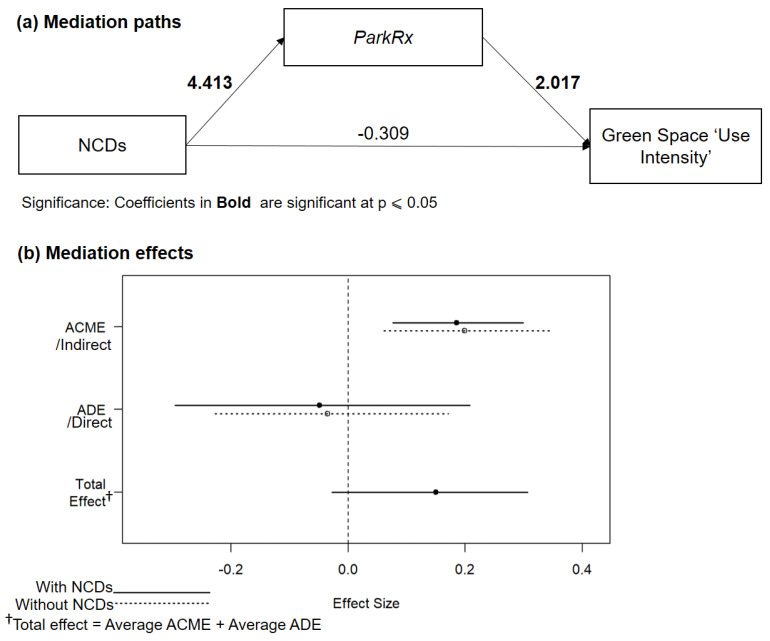
(**a**) ParkRx as a mediator of the NCDs and use intensity association. (**b**) Average causal mediation (ACME), average direct effect (ADE) for both NCDs and non-NCDs groups, and the total effect of NCDs and ParkRx on ‘Use intensity’.

Model 4 (mediator model) showed that NCDs strongly predict ParkRx*,* which, in turn, predicts urban green space use (Figure 3a). Respondents with NCDs were 82 times more likely to report park prescriptions than other respondents (Table A1, Appendix A). 

Model 5 (outcome model) provides further evidence that ParkRx mediates the relationship between NCDs and green space use. ParkRx remained statistically significant in the logistic regression when ParkRx and NCDs were included, whereas NCDs were not statistically significant in Model 5 (Table A2). Because we found that NCDs were positively associated with use intensity when the ParkRx variable was not included (see Model 2 in Table 5), the results of Model 5 in Table A2 support the conclusion that ParkRx mediates the direct relationship between NCDs and use intensity.

This conclusion was also supported in the model effects. The average causal mediation effect (ACME) was positive and significant (Figure 3b). The average direct effect (ADE) was negative and non-significant for all respondents (Table A3). The average total effect was positive and marginally significant (*p* < 0.10). The findings indicated that the ACME had a greater total effect on use intensity than the ADE. The ACME value was 0.192, *p* < 0.05 (see Table 6), indicating that the expected mediation effect (H1) was supported for all the respondents irrespective of NCD status. Also, the ADE was near zero and non-significant, and the proportion mediated value was greater than one, showing that the presence of a ParkRx completely mediated the direct relationship between NCDs and green space use intensity. However, the 95% confidence interval for the proportion mediated included zero (−0.239 to 8.1, Table 6); therefore, the complete mediation results need to be interpreted with caution, as, in some cases, the proportion mediated can be less than one, indicating a partial mediation effect of ParkRx on the direct relationship between NCDs and green space use intensity.

## 4. Discussion

### 4.1. General Findings

To our knowledge, this is the first study to test whether noncommunicable diseases and ParkRx influence green space use in a Global South megacity. Our case study provides new evidence regarding what factors motivate middle-aged adults to visit green spaces and access the health benefits of exposure to natural environments in Dhaka, Bangladesh. 

Our findings are not aligned with past work on the importance of green space attractiveness on visitation [30,68,76,78]. In Dhaka, quality and attractiveness did not impact how often high-intensity users visited green spaces or how long they stayed there, which is perhaps due to the limited availability and quality of green spaces in the city [59]. Most of our respondents did not indicate that the green spaces they visited were particularly attractive, which is likely a consequence of the improper management and upkeep of parks in Dhaka [63]. These findings highlight that, for our case study city in the Global South, where the supply of green spaces is often lower than demand [57,63], even poor-quality green spaces are used intensively.

Our findings that show no significant associations between green space accessibility and use are consistent with some studies [77,80,110], but not others [31,34,68,75,111]. One potential reason for our findings might be the limited range of our data. At present, the provision of green spaces in Dhaka is minimal, and most of our respondents indicated poor accessibility to green spaces. Due to the low accessibility level for most of the respondents, there was a lack of variation in our data regarding accessibility between low- and high-intensity users. Another explanation for these results could be our approach of measuring accessibility; we used subjective assessment (e.g., perceived access), whereas some studies that utilized objective measurement (e.g., distance to green space from home) observed significant associations [34,68,81,112]. 

In addition to accessibility, we observed that neighborhood-level green space availability is not associated with use intensity. This finding is not aligned with previous studies indicating that green space availability at the neighborhood level is associated with green space use [92,93,110,111,113]. We argue that, as with the lack of variations in green space accessibility, the overall low availability of green space in Dhaka is responsible for the non-significant association between green space availability and ‘Use intensity.’ Additionally, studies with contrasting results focused on Global North contexts, where the availability of green space at the neighborhood level is usually higher than in Dhaka [57].

Our case study is one of the few studies that investigated how attachment to green space influences use intensity among middle-aged adults. The results indicated that attachment to green space significantly increased the chance of high-intensity use. Our findings are consistent with several previous studies, where researchers also found that attachment to natural spaces was associated with increased usage [85,87,88,90]. Our findings are also consistent with Budrick et al.’s [91] study set in another Global South city (Pune, India), who reported that place identity and place attachment were associated with pro-environmental attitudes among urban green space users. We explain green space attachment and increased usage based on the level of social capital and social support associated with green space and health [91,114,115,116]. In our study, respondents visiting the same green space for a greater number of years were more familiar with the local environment than other people visiting the green space for a shorter number of years. Familiarity with the local environment might induce place identity and increase users’ perceived safety of the green space [90,91,115]. Additionally, regular visits to green space for longer periods have the potential to help group formation (e.g., diabetic patient walker groups and friendships), which provides green space users with opportunities for social participation. These factors encourage more frequent and longer green space visits. Recently, Gaikwad and Shinde [38] also observed a similar phenomenon among older adults when studying park usage in Pune, India. 

Our findings related to attractiveness, accessibility, and availability of green space and use intensity contrast with several other case studies [31,34,68,78,93]. Green space use intensity among middle-aged residents in Dhaka might work in ways different from what has been observed in studies set in Global North contexts. In this regard, the analysis of NCDs and ParkRx provides key insights regarding green space use intensity in Dhaka.

One of the unique aspects of this study was the introduction of poor health condition and ParkRx as predictors of green space usage in our Dhaka case study set in the Global South context. The results indicated that in Dhaka, the middle-age respondents with NCDs are more likely to be high-intensity urban green space users than respondents without NCDs (Model 2). We also found that respondents with a ParkRx are more likely to exhibit high-intensity use compared to respondents without a ParkRx (Model 3). Both variables are crucial in understanding urban green space usage in the context of Dhaka. With increasing noncommunicable disease burdens in Dhaka and the overall Global South [96,97,117,118], more people are being diagnosed with NCDs, such as diabetes and cardiovascular disease. Healthcare professionals often recommend outdoor physical exercise for such patients in addition to regular medications, resulting in more patients engaging in health promotion in green spaces through active (moderate to vigorous physical activity) and passive activities (social gatherings, nature viewing). Our finding that the high-intensity middle-aged users of green spaces in Dhaka usually have NCDs and are prescribed ParkRx supports this pathway between poor health and green space use. 

The NCD and ParkRx results help explain why our other findings on green space attractiveness, accessibility, and availability do not completely align with those in the literature, and thus indicate case-specific differences in urban green space use in the context of a Global South megacity (i.e., Dhaka). Indeed, even if the respondents found the green spaces unattractive, with poor access, and with overall low availability, the high-intensity green space users were motivated by their health conditions and by a doctor’s prescription.

Our study’s main contribution to the growing literature on the green space–health linkages is the statistically significant mediation effect of ParkRx on the relationship between NCDs and green space use intensity. The mediation analysis allowed us to determine whether an NCD diagnosis or a ParkRx could explain whether an individual would exhibit high or low green space use intensity. Although both NCDs and ParkRx had positive associations with green space use intensity (Models 2 and 3), the mediation analysis indicated that ParkRx was the most important underlying reason for a middle-aged adult’s use of urban green spaces in Dhaka. Our findings were consistent with a few several previous studies on ParkRx conducted in the Global North [35,39,41,48]. However, previous studies only identified the direct relationship between ParkRx and park or green space usage, whereas our results showed both the direct and mediation effects of ParkRx on green space usage. 

### 4.2. Policy and Practice Implications 

The results of the present study may help healthcare professionals, policymakers, and urban planners grasp the importance of urban green space and ParkRx in reducing the burden of NCDs in Dhaka and, to some extent, other Global South cities that share similarities with Dhaka. Because Dhaka has a supply deficiency of quality green spaces for its population and because of the documented benefits of green space supply in other settings [6,119], the city requires an aggressive urban greening policy to engender a proactive approach to linking health with the environment and supporting outdoor physical exercise. Currently, the urban planning policies in the city are inadequate and do not provide the minimum required per capita green spaces as recommended by the WHO [57,120]. Approaches such as ‘compensative greening’ [120], and ‘redevelopment and resettlement’ [121] should be considered to push greening initiatives in Dhaka and other Global South cities forward. In Dhaka, a few park revitalization programs (e.g., Jol-Sobujer Dhaka Project) have recently been implemented [122], and the Dhaka City Neighborhood Upgrading Project (106.00 million USD, 2019−2024), funded by the World Bank, is planning to increase open spaces over the next four years [123]. Even if our findings show that green space attractiveness is not associated with higher use intensity, they do not exonerate Dhaka from its mandate to create well-maintained green spaces for all residents regardless of socioeconomic status and race/ethnicity. Such initiatives, when they focus on disadvantaged neighborhoods, can help reverse environmental injustices in access to urban green space in Global South cities [23], which echo injustices in Global North contexts [5,124,125]. However, urban planners and elected officials need to ensure that such greening initiatives in disadvantaged neighborhoods do not result in environmental gentrification like in Global North cities, which involves the influx of wealthier residents to low-income neighborhoods due, in part, to new green amenities, as well as the eventual displacement of poor residents [125,126].

Furthermore, our findings suggest that healthcare professionals should consider ParkRx as an important treatment option for NCD patients. Our empirical evidence showed that ParkRx was linked to high-intensity use of green spaces among respondents with NCDs; however, not all NCD patients in our sample received a ParkRx. Considering the benefits of physical exercise in green spaces [1,119,127], we suggest that healthcare professionals should increasingly promote ParkRx to their patients and conduct regular follow-ups to reduce the burden of cardiovascular disease, diabetes, and other NCDs. Notably, ParkRx seems to be critical for middle-aged adults in Dhaka, and it might also be useful in other Global South megacities, as middle-aged adults are more frequently diagnosed with NCDs [50,51]. Recommending ParkRx to newly diagnosed patients may promote healthy living by increasing their physical exercise in green spaces and reducing the need for additional medications. In the long term, the presence and use of urban green space might alleviate burdens on healthcare systems with physicians prescribing ParkRx through reduced doctor visits and incurred costs [128,129,130]. 

### 4.3. Strengths, Limitations, and Future Directions

The strengths of this study include its consideration of urban green space use patterns among middle-aged residents in a Global South megacity (i.e., Dhaka), its focus on health condition (i.e., NCDs) and ParkRx, and our mediation analysis. Our findings add new insights to the literature regarding the factors influencing urban green space use in the case of Dhaka and how these factors are consistent with or different from results in other case studies. Importantly, our mediation analysis shows a potential pathway between an NCD diagnosis, a ParkRx, and use intensity of green space. 

We acknowledge that this study has limitations. First, due to the unavailability of residential addresses, we could not objectively measure the accessibility of green spaces; however, other studies indicated that subjective or perceived accessibility is a more pronounced indicator of actual green space use than objective accessibility [82,83]. Second, due to resource and data limitations, we were unable to consider variables such as land use mix, walkability, perceived air and noise pollution, and perceived safety [19,119]. These variables could also influence green space use patterns in Dhaka. Third, although our mediation analysis suggested a causal effect of ParkRx on green space use intensity after 1000 simulations, these findings were based on cross-sectional observations. Therefore, we cannot conclude that the associations we observed in our models indicate causal inference. Fourth, the health condition and ParkRx variables were self-reported, and our study design did not involve control and treatment groups; thus, there might be other unmeasured confounders impacting green space use intensity. Finally, we only considered a relatively small sample of middle-aged residents in a single case study city. Green space use may differ considerably among different age groups, such as children and older adults, and in different geographic contexts.

Considering these limitations, we recommend the following directions for future research. First, similar studies on ParkRx should be replicated in other Global South megacities (e.g., Delhi, Accra, Addis Ababa) to clarify whether our findings can be extended to other contexts and to understand the broader implications of green space in urban planning and health promotion. Second, additional environmental (e.g., air and noise pollution) and socioeconomic variables should be integrated into models of green space use in the context of Dhaka and other Global South megacities. Third, researchers should conduct randomized control trials with longitudinal designs to identify the causal effects of health condition and ParkRx on green space use intensity. Finally, future work in Dhaka and other Global South megacities could use more advanced mediational analyses, such as differences-in-difference techniques, to examine the impact of green space quality on green space use [105], taking advantage of natural experiment opportunities offered by the construction of new high-quality parks.

## 5. Conclusions

In this study, we investigated the factors associated with green space use intensity among middle-aged adults, taking into account their health conditions and park prescriptions. Most previous green space and health studies have focused on cities in the Global North, and limited evidence is available regarding the predictors of green space use in megacities in the Global South. We provide new evidence for this body of literature by setting this study in Dhaka City, an emerging megacity in the Global South. 

Our analyses provided suggestive evidence of strong positive associations between health condition, ParkRx, and green space use intensity. We observed that people with non-communicable diseases and a green space prescription (ParkRx) are usually high-intensity urban green space users. Notably, we identified a statistically significant mediation effect of ParkRx on the association between health condition and green space use intensity. This suggests that ParkRx may explain the underlying mechanisms of the use of green space to improve health. Thus, we conclude that poor health condition and healthcare professionals’ recommendations for being outdoors and engaging in physical exercise and other health-promoting activities (such as is done with ParkRx) might be the key to understanding the green space use intensity among middle-aged adults in Dhaka. Our findings have implications for urban greening, public health policies, and healthcare professionals’ practice for Dhaka and, to some extent, for other Global South megacities to reduce noncommunicable disease burdens through the use of urban green spaces. 

## Figures and Tables

**Table 1:** Generation of the binary omnibus measure of green space use.

	Frequency		
Duration		Low	High
	Low	Low-frequency low-duration	High-frequency low-duration
	High	Low-frequency high-duration	High-frequency high-duration

**Table 2:** Sample characteristics.

Attribute	Categories	Total Sample, 169	
		N	Percentage
Gender	Female	65	38.5
	Male	104	61.5
Education	None	52	30.8
	Secondary School	42	24.9
	Higher Secondary School	28	16.6
	Bachelors	11	6.5
	Post-Graduate (MSc/PhD)	36	21.2
Employment	Unemployed	57	33.7
	Retired	14	8.3
	Employed	98	58
Travel Mode	Walk	134	79.3
	Bicycle or motorcycle	10	5.9
	Public transport (e.g., bus)	14	8.3
	Private vehicle	6	3.6
	Others (e.g., rickshaw)	5	3.0
NCDs	No	50	29.6
	Yes	119	70.4
Park prescription (ParkRx)	No	81	47.9
	Yes	88	52.1

**Table 3:** Green space characteristics and use patterns among middle-aged residents in Dhaka.

Measurements	Categories	N	Percentage
Perceived distance (*accessibility*)	Less than 5 min	11	6.5
	5 to 10 min	67	39.6
	11 to 15 min	45	26.6
	16 to 20 min	31	18.3
	More than 20 min	15	8.9
Perceived attractiveness	Very low	14	8.3
	Low	44	26.0
	Moderate	47	27.8
	High	31	18.3
	Very high	33	19.5
Years using the green space (*attachment*)	<=1 year	74	43.8
	2−3 years	47	27.8
	3−4 years	29	17.2
	4−5 years	7	4.1
	>5 years	12	7.1
Visit *frequency* (usual week)	Less or equal 4 days	46	27.2
	More than 5 days	123	72.8
Visit *duration* (min/visit on average)	Less than 30 min	99	58.6
	More than 30 min	70	41.4
*Use intensity* (omnibus measure from frequency and duration values)	Low	111	65.7
	High	58	34.3

**Table 4:** Green space use variations among noncommunicable diseases (NCDs) and park prescription (ParkRx) groups.

			**ParkRx**		
			**No**	**Yes**	**Total Count**
Do you have NCDs/chronic health issue(s)?	No	Count	48	2	50
		% within NCDs	96.0%	4.0%	
		% within ParkRx	59.3%	2.3%	
	Yes	Count	33	86	119
		% within NCDs	27.7%	72.3%	
		% within ParkRx	40.7%	97.7%	
	Total Count		81	88	169
			**Green Space ‘Use Intensity’**		
			**Low**	**High**	**Total Count**
Do you have NCDs/chronic health issue(s)?	No	Count	40	10	50
		% within NCDs	80.0%	20.0%	
		% within use Intensity	36.0%	17.2%	
	Yes	Count	71	48	119
		% within NCDs	59.7%	40.3%	
		% within use Intensity	64.0%	82.8%	
	Total Count		111	58	169

**Table 5:** Associations of green space characteristics, chronic disease condition, and ParkRx with use intensity.

	Model 1				Model 2			Model 3			
	Odds	95% CI	*p*-value		Odds	95% CI	*p*-value	Odds		95% CI	*p*-value
Attractiveness	**0.712**	0.52–0.959	0.0288		**0.713**	0.518–0.965	0.032	0.735		0.524–1.015	0.0667
Accessibility	0.824	0.568–1.187	0.3015		0.795	0.542–1.156	0.2327	0.75		0.501–1.108	0.1531
Attachment	**2.095**	1.528–2.972	0.0000114		**2.004**	1.453–2.851	0.0000469	**2.08**		1.486–3.013	0.0000429
Green space per capita	1.032	0.974–1.212	0.404		1.028	0.95–1.109	0.4719	1.011		0.93–1.095	0.775
NCDs	-	-			**2.821**	1.148–7.573	0.0296	-		-	-
ParkRx	-	-			-	-		**6.328**		2.706–16.075	0.0000438
Constant	0.292	0.025–3.135	0.3147		0.119	0.008–1.512	0.1077	**0.048**		0.003–0.667	0.0264
Pseudo R^2^	0.228			0.263					0.353		
AIC	204.86			201.68					187.42		

CI = confidence interval of odds (lower–upper). Odds ratios in **Bold** are significant at *p* ≤ 0.05. Underlined values are significant at *p* ≤ 0.10. All models are adjusted for gender, education, employment, and travel mode.

**Table 6:** Mediation analyses of NCDs and mediator ParkRx on use intensity.

	Estimate	95% CI Lower	95% CI Upper	*p*-Value
ACME (average)	**0.192**	0.0696	0.32	0.002
ADE (average)	−0.042	−0.2583	0.19	0.712
Total Effect	0.15	−0.028	0.31	0.076
Prop. Mediated	1.228	−2.3953	8.1	0.078

ACME = average causal mediation effect, ADE = average direct effect, Prop.Mediated = proportion mediated. CI = confidence interval. Coefficients in **Bold** are significant at *p* ≤ 0.05. Underlined values are significant at *p* ≤ 0.10.

## Appendix A

**Table A1:** Mediator model results—associations of NCDs with ParkRx (Model 4).

	Model 4 †		
	Odds	95% CI	*p*-Value
Attractiveness	0.726	0.503–1.031	0.0788
Accessibility	1.159	0.746–1.809	0.5093
Attachment	1.222	0.843–1.799	0.2945
Green space per capita	**1.164**	1.03–1.357	0.029
NCDs	**82.589**	19.524−711.112	0.000000439
Constant	0.232	0.007–5.801	0.3842
Pseudo R^2^	0.57		
AIC	158.85		

CI = confidence interval of odds (lower–upper). Odds ratios in **Bold** are significant at *p* ≤ 0.05. Underlined values are significant at *p* ≤ 0.10. †: Model is adjusted for gender, education, employment, and travel mode.

**Table A2:** Outcome model for mediation analysis (Model 5).

	Model 5 †		
	Odds	95% CI	*p*-Value
Attractiveness	0.734	0.522–1.015	0.0665
Accessibility	0.756	0.505–1.121	0.1688
Attachment	**2.104**	1.497–3.062	0.0000401
Green space per capita	1.01	0.92–1.09	0.7972
NCDs/Chronic	0.733	0.202–2.543	0.6273
ParkRx	**7.519**	2.554–25.663	0.000539
Constant	**0.051**	0.003–0.736	0.031588
Pseudo R^2^	0.355		
AIC	189.18		

CI = confidence interval of odds (lower–upper). Odds ratios in **Bold** are significant at *p* ≤ 0.05. Underlined values are significant at *p* ≤ 0.10. †: Model is adjusted for gender, education, employment, and travel mode.

**Table A3:** Mediation results of all effects.

	Estimate	95% CI Lower	95% CI Upper	*p*-Value
ACME (without NCDs)	**0.1991**	0.061	0.35	0.002
ACME (with NCDs)	**0.1852**	0.0762	0.3	0.002
ADE (without NCDs)	−0.0352	−0.2273	0.17	0.712
ADE (with NCDs)	−0.0491	−0.2957	0.21	0.712
Total Effect	0.15	−0.028	0.31	0.076
Prop. Mediated (without NCDs)	1.2626	−2.6145	8.83	0.078
Prop. Mediated (with NCDs)	1.1933	−2.0749	7.68	0.078
ACME (average)	**0.1921**	0.0696	0.32	0.002
ADE (average)	−0.0421	−0.2583	0.19	0.712
Prop. Mediated	1.228	−2.3953	8.1	0.078

ACME = Average causal mediation effect, ADE = Average direct effect, Prop.Mediated = Proportion mediated. CI = confidence interval of odds. Coefficients in **Bold** are significant at *p* ≤ 0.05. Underlined values are significant at *p* ≤ 0.10.

## References

[1] Kondo M.C., Fluehr J.M., McKeon T., Branas C.C. (2018). Urban green space and its impact on human health. Int. J. Environ. Res. Public Health.

[2] Leng H., Li S., Yan S., An X. (2020). Exploring the relationship between green space in a neighbourhood and cardiovascular health in the winter city of China: A study using a health survey for harbin. Int. J. Environ. Res. Public Health.

[3] Schinasi L.H., Quick H., Clougherty J.E., De Roos A.J. (2019). Greenspace and Infant Mortality in Philadelphia, PA. J. Urban Heal..

[4] Astell-Burt T., Feng X., Kolt G.S. (2014). Greener neighborhoods, slimmer people evidence from 246 920 Australians. Int. J. Obes..

[5] Mitchell R., Popham F. (2008). Effect of exposure to natural environment on health inequalities: An observational population study. Lancet.

[6] Markevych I., Schoierer J., Hartig T., Chudnovsky A., Hystad P., Dzhambov A.M., de Vries S., Triguero-Mas M., Brauer M., Nieuwenhuijsen M.J. (2017). Exploring pathways linking greenspace to health: Theoretical and methodological guidance. Environ. Res..

[7] Kabisch N., Qureshi S., Haase D. (2015). Human-environment interactions in urban green spaces—A systematic review of contemporary issues and prospects for future research. Environ. Impact Assess. Rev..

[8] James P., Banay R.F., Hart J.E., Laden F. (2015). A Review of the Health Benefits of Greenness. Curr. Epidemiol. Reports.

[9] Lindley S.J., Cook P.A., Dennis M., Gilchrist A. (2019). Biodiversity, Physical Health and Climate Change: A Synthesis of Recent Evidence. Biodiversity and Health in the Face of Climate Change.

[10] Bezold C.P., Stark J.H., Rundle A., Konty K., Day S.E., Quinn J., Neckerman K., Roux A.V.D. (2017). Relationship between Recreational Resources in the School Neighborhood and Changes in Fitness in New York City Public School Students. J. Urban Heal..

[11] Chodzko-Zajko W., Schwingel A. (2009). Chae Hee Park Successful Aging: The Role of Physical Activity. Am. J. Lifestyle Med..

[12] Astell-Burt T., Feng X., Kolt G.S. (2014). Green space is associated with walking and moderate-to-vigorous physical activity (MVPA) in middle-to-older-aged adults: Findings from 203 883 Australians in the 45 and Up Study. Br. J. Sports Med..

[13] Rojas-Rueda D., Nieuwenhuijsen M.J., Gascon M., Perez-Leon D., Mudu P. (2019). Green spaces and mortality: A systematic review and meta-analysis of cohort studies. Lancet Planet. Heal..

[14] Browning M.H.E.M., Rigolon A. (2019). School Green Space and Its Impact on Academic Performance: A Systematic Literature Review. Int. J. Environ. Res. Public Health.

[15] Dzhambov A.M., Markevych I., Hartig T., Tilov B., Arabadzhiev Z., Stoyanov D., Gatseva P., Dimitrova D.D. (2018). Multiple pathways link urban green- and bluespace to mental health in young adults. Environ. Res..

[16] 1 Maas J., van Dillen S.M.E., Verheij R.A., Groenewegen P.P. (2009). Social contacts as a possible mechanism behind the relation between green space and health. Heal. Place.

[17] Dadvand P., Nieuwenhuijsen M.J., Esnaola M., Forns J., Basagaña X., Alvarez-Pedrerol M., Rivas I., López-Vicente M., De Pascual M.C., Su J. (2015). Green spaces and cognitive development in primary schoolchildren. Proc. Natl. Acad. Sci. USA.

[18] World Economic Situation and Prospects (WESP) 2017 | Multimedia Library—United Nations Department of Economic and Social Affairs. https://www.un.org/development/desa/publications/world-economic-situation-and-prospects-wesp-2017.html.

[19] Labib S.M., Lindley S., Huck J.J. (2020). Spatial dimensions of the influence of urban green-blue spaces on human health: A systematic review. Environ. Res..

[20] Cleland C., Reis R.S., Ferreira Hino A.A., Hunter R., Fermino R.C., de Paiva H.K., Czestschuk B., Ellis G. (2019). Built environment correlates of physical activity and sedentary behaviour in older adults: A comparative review between high and low-middle income countries. Heal. Place.

[21] Schetke S., Qureshi S., Lautenbach S., Kabisch N. (2016). What determines the use of urban green spaces in highly urbanized areas? - Examples from two fast growing Asian cities. Urban For. Urban Green..

[22] Van Cauwenberg J., Nathan A., Barnett A., Barnett D.W., Cerin E. (2018). Relationships Between Neighbourhood Physical Environmental Attributes and Older Adults’ Leisure-Time Physical Activity: A Systematic Review and Meta-Analysis. Sport. Med..

[23] Rigolon A., Browning M., Lee K., Shin S. (2018). Access to Urban Green Space in Cities of the Global South: A Systematic Literature Review. Urban Sci..

[24] Nagendra H., Bai X., Brondizio E.S., Lwasa S. (2018). The urban south and the predicament of global sustainability. Nat. Sustain..

[25] Braimoh A.K., Onishi T. (2007). Spatial determinants of urban land use change in Lagos, Nigeria. Land Use Policy.

[26] Dewan A.M., Yamaguchi Y. (2009). Land use and land cover change in Greater Dhaka, Bangladesh: Using remote sensing to promote sustainable urbanization. Appl. Geogr..

[27] Zérah M.H. (2007). Conflict between green space preservation and housing needs: The case of the Sanjay Gandhi National Park in Mumbai. Cities.

[28] Ahmed B., Ahmed R. (2012). Modeling urban land cover growth dynamics using multioral satellite images: A case study of Dhaka, Bangladesh. ISPRS Int. J. Geo-Inf..

[29] Labib S.M., Neema M.N., Rahaman Z., Patwary S.H., Shakil S.H. (2018). Carbon dioxide emission and bio-capacity indexing for transportation activities: A methodological development in determining the sustainability of vehicular transportation systems. J. Environ. Manag..

[30] McCormack G.R., Rock M., Toohey A.M., Hignell D. (2010). Characteristics of urban parks associated with park use and physical activity: A review of qualitative research. Heal. Place.

[31] Wang D., Brown G., Zhong G., Liu Y., Mateo-Babiano I. (2015). Factors influencing perceived access to urban parks: A comparative study of Brisbane (Australia) and Zhongshan (China). Habitat Int..

[32] Zhang W., Yang J., Ma L., Huang C. (2015). Factors affecting the use of urban green spaces for physical activities: Views of young urban residents in Beijing. Urban For. Urban Green..

[33] Razani N., Morshed S., Kohn M.A., Wells N.M., Thompson D., Alqassari M., Agodi A., Rutherford G.W. (2018). Effect of park prescriptions with and without group visits to parks on stress reduction in low-income parents: SHINE randomized trial. PLoS ONE.

[34] Schipperijn J., Cerin E., Adams M.A., Reis R., Smith G., Cain K., Christiansen L.B., Dyck D., van Gidlow C., Frank L.D. (2017). Access to parks and physical activity: An eight country comparison. Urban For. Urban Green..

[35] Müller-Riemenschneider F., Petrunoff N., Yao J., Ng A., Sia A., Ramiah A., Wong M., Han J., Tai B.C., Uijtdewilligen L. (2020). Effectiveness of prescribing physical activity in parks to improve health and wellbeing-the park prescription randomized controlled trial. Int. J. Behav. Nutr. Phys. Act..

[36] Yigitcanlar T., Kamruzzaman M., Teimouri R., Degirmenci K., Alanjagh F. (2020). Association between park visits and mental health in a developing country context: The case of Tabriz, Iran. Landsc. Urban Plan..

[37] Nath T.K., Zhe Han S.S., Lechner A.M. (2018). Urban green space and well-being in Kuala Lumpur, Malaysia. Urban For. Urban Green..

[38] Gaikwad A., Shinde K. (2019). Use of parks by older persons and perceived health benefits: A developing country context. Cities.

[39] Patel A., Schofield G.M., Kolt G.S., Keogh J.W. (2011). General practitioners’ views and experiences of counselling for physical activity through the New Zealand Green Prescription program. BMC Fam. Pract..

[40] Sefcik J.S., Kondo M.C., Klusaritz H., Sarantschin E., Solomon S., Roepke A., South E.C., Jacoby S.F. (2019). Perceptions of nature and access to green space in four urban neighborhoods. Int. J. Environ. Res. Public Health.

[41] Razani N., Hills N.K., Thompson D., Rutherford G.W. (2020). The association of knowledge, attitudes and access with park use before and after a park-prescription intervention for low-income families in the U.S. Int. J. Environ. Res. Public Health.

[42] Seltenrich N. (2015). Just What the Doctor Ordered: Using Parks to Improve Children’s Health. Environ. Health Perspect..

[43] James J.J., Christiana R.W., Battista R.A. (2019). A historical and critical analysis of park prescriptions. J. Leis. Res..

[44] Robinson J., Breed M. (2019). Green Prescriptions and Their Co-Benefits: Integrative Strategies for Public and Environmental Health. Challenges.

[45] Mnich C., Weyland S., Jekauc D., Schipperijn J. (2019). Psychosocial and physiological health outcomes of green exercise in children and adolescents—A systematic review. Int. J. Environ. Res. Public Health.

[46] Crnic M., Kondo M.C. (2019). Nature RX: Reemergence of pediatric nature-based therapeutic programs from the late 19th and early 20th centuries. Am. J. Public Health.

[47] Zarr R., Cottrell L., Merrill C. (2017). Park prescription (DC Park Rx): A new strategy to combat chronic disease in children. J. Phys. Act. Heal..

[48] Razani N., Niknam K., Wells N.M., Thompson D., Hills N.K., Kennedy G., Gilgoff R., Rutherford G.W. (2019). Clinic and park partnerships for childhood resilience: A prospective study of park prescriptions. Heal. Place.

[49] BBC “Nature” Being Prescribed by GPs in Shetland—BBC News. https://www.bbc.co.uk/news/uk-scotland-north-east-orkney-shetland-45758016.

[50] Noncommunicable Diseases. https://www.who.int/news-room/fact-sheets/detail/noncommunicable-diseases.

[51] Ghaffar A., Reddy K.S., Singhi M. (2002). Burden of non-communicable diseases in South Asia. BMJ.

[52] Ahmed S., Nahiduzzaman K.M., Hasan M.M.U. (2018). Dhaka, Bangladesh: Unpacking challenges and reflecting on unjust transitions. Cities.

[53] Labib S.M., Harris A. (2018). The potentials of Sentinel−2 and LandSat−8 data in green infrastructure extraction, using object based image analysis (OBIA) method. Eur. J. Remote Sens..

[54] Kumar P., Druckman A., Gallagher J., Gatersleben B., Allison S., Eisenman T.S., Hoang U., Hama S., Tiwari A., Sharma A. (2019). The nexus between air pollution, green infrastructure and human health. Environ. Int..

[55] Bloemsma L.D., Wijga A.H., Klompmaker J.O., Janssen N.A.H., Smit H.A., Koppelman G.H., Brunekreef B., Lebret E., Hoek G., Gehring U. (2019). The associations of air pollution, traffic noise and green space with overweight throughout childhood: The PIAMA birth cohort study. Environ. Res..

[56] Labib S.M., Mohiuddin H., Hasib I.M.A., Sabuj S.H., Hira S. (2019). Integrating Data Mining and Microsimulation Modelling to Reduce Traffic Congestion: A Case Study of Signalized Intersections in Dhaka, Bangladesh. Urban Sci..

[57] Rahman K.M.A., Zhang D. (2018). Analyzing the level of accessibility of public urban green spaces to different socially vulnerable groups of people. Sustain..

[58] Labib S.M. (2017). Volunteer GIS (VGIS) Based Waste Management A conceptual design and use of Web 2.0 for Smart Waste Management in Dhaka City. Third International Conference on Research in Computational Intelligence and Communication Networks (ICRCICN).

[59] Byomkesh T., Nakagoshi N., Dewan A.M. (2012). Urbanization and green space dynamics in Greater Dhaka, Bangladesh. Landsc. Ecol. Eng..

[60] About Parks: NYC Parks. https://www.nycgovparks.org/about.

[61] Comber A., Brunsdon C., Green E. (2008). Using a GIS-based network analysis to determine urban greenspace accessibility for different ethnic and religious groups. Landsc. Urban Plan..

[62] 10minutewalk | The Trust for Public Land. https://www.tpl.org/10minutewalk.

[63] Tabassum S., Sharmin F. (2013). Accessibility Analysis of Parks at Urban Neighborhood: The Case of Dhaka.

[64] Shanahan D.F., Bush R., Gaston K.J., Lin B.B., Dean J., Barber E., Fuller R.A. (2016). Health Benefits from Nature Experiences Depend on Dose. Sci. Rep..

[65] Cleary A., Roiko A., Burton N.W., Fielding K.S., Murray Z., Turrell G. (2019). Changes in perceptions of urban green space are related to changes in psychological well-being: Cross-sectional and longitudinal study of mid-aged urban residents. Heal. Place.

[66] Astell-Burt T., Feng X., Kolt G.S. (2013). Mental health benefits of neighbourhood green space are stronger among physically active adults in middle-to-older age: Evidence from 260,061 Australians. Prev. Med..

[67] Hunter R.F., Christian H., Veitch J., Astell-Burt T., Hipp J.A., Schipperijn J. (2015). The impact of interventions to promote physical activity in urban green space: A systematic review and recommendations for future research. Soc. Sci. Med..

[68] Akpinar A. (2016). How is quality of urban green spaces associated with physical activity and health?. Urban For. Urban Green..

[69] Shuvo F.K. (2020). Do the Features of Urban Green Spaces that Promote Social and Active Ageing Vary by International Context? Comparison between and within Sydney, Dhaka and Singapore.

[70] Khan M. (2014). Study of Open Spaces in the Context of Dhaka City for Sustainable Use: A Syntactic Approach. Int. J. Eng. Technol..

[71] Siddiqui N.I., Nessa A., Hossain M.A. (2010). Regular physical exercise: Way to healthy life. Mymensingh Med. J..

[72] Piercy K.L., Troiano R.P., Ballard R.M., Carlson S.A., Fulton J.E., Galuska D.A., George S.M., Olson R.D. (2018). The physical activity guidelines for Americans. JAMA J. Am. Med. Assoc..

[73] JMR G., AR C. (2008). Physical activity and prevention of type 2 diabetes mellitus. Sport. Med..

[74] Hillsdon M., Panter J., Foster C., Jones A. (2006). The relationship between access and quality of urban green space with population physical activity. Public Health.

[75] Giles-Corti B., Broomhall M.H., Knuiman M., Collins C., Douglas K., Ng K., Lange A., Donovan R.J. (2005). Increasing walking: How important is distance to, attractiveness, and size of public open space?. Am. J. Prev. Med..

[76] Sugiyama T., Francis J., Middleton N.J., Owen N., Giles-CortI B. (2010). Associations between recreational walking and attractiveness, size, and proximity of neighborhood open spaces. Am. J. Public Health.

[77] Koohsari M.J., Kaczynski A.T., Giles-Corti B., Karakiewicz J.A. (2013). Effects of access to public open spaces on walking: Is proximity enough?. Landsc. Urban Plan..

[78] Brindley P., Cameron R.W., Ersoy E., Jorgensen A., Maheswaran R. (2019). Is more always better? Exploring field survey and social media indicators of quality of urban greenspace, in relation to health. Urban For. Urban Green..

[79] Ekkel E.D., de Vries S. (2017). Nearby green space and human health: Evaluating accessibility metrics. Landsc. Urban Plan..

[80] Kaczynski A.T., Besenyi G.M., Stanis S.W.A., Koohsari M.J., Oestman K.B., Bergstrom R., Potwarka L.R., Reis R.S. (2014). Are park proximity and park features related to park use and park-based physical activity among adults? Variations by multiple socio-demographic characteristics. Int. J. Behav. Nutr. Phys. Act..

[81] Shwe Zin Nyunt M., Shuvo F.K., Yen Eng J., Bee Yap K., Scherer S., Min Hee L., Pang Chan S., Pin Ng T. (2015). Objective and subjective measures of neighborhood environment (NE): Relationships with transportation physical activity among older persons. Int. J. Behav. Nutr. Phys. Act..

[82] Giles-Corti B., Donovan R.J. (2002). Socioeconomic status differences in recreational physical activity levels and real and perceived access to a supportive physical environment. Prev. Med..

[83] Hoehner C.M., Brennan Ramirez L.K., Elliott M.B., Handy S.L., Brownson R.C. (2005). Perceived and objective environmental measures and physical activity among urban adults. Am. J. Prev. Med..

[84] Cerin E., Leslie E., Owen N., Bauman A. (2008). An Australian version of the neighborhood environment walkability scale: Validity evidence. Meas. Phys. Educ. Exerc. Sci..

[85] Lee T.H., Shen Y.L. (2013). The influence of leisure involvement and place attachment on destination loyalty: Evidence from recreationists walking their dogs in urban parks. J. Environ. Psychol..

[86] Lewicka M. (2011). Place attachment: How far have we come in the last 40 years?. J. Environ. Psychol..

[87] Clark W.A.V., Duque-Calvache R., Palomares-Linares I. (2017). Place Attachment and the Decision to Stay in the Neighbourhood. Popul. Space Place.

[88] Ryan R.L. (2006). The role of place attachment in sustaining urban parks. The Humane Metropolis: People and Nature in the 21st-Century City.

[89] Lewicka M. (2005). Ways to make people active: The role of place attachment, cultural capital, and neighborhood ties. J. Environ. Psychol..

[90] Arnberger A., Eder R. (2012). The influence of green space on community attachment of urban and suburban residents. Urban For. Urban Green..

[91] Budruk M., Thomas H., Tyrrell T. (2009). Urban green spaces: A study of place attachment and environmental attitudes in India. Soc. Nat. Resour..

[92] Triguero-Mas M., Donaire-Gonzalez D., Seto E., Valentín A., Smith G., Martínez D., Carrasco-Turigas G., Masterson D., van den Berg M., Ambròs A. (2017). Living close to natural outdoor environments in four European cities: Adults’ contact with the environments and physical activity. Int. J. Environ. Res. Public Health.

[93] Maas J., Verheij R.A., Groenewegen P.P., De Vries S., Spreeuwenberg P. (2006). Green space, urbanity, and health: How strong is the relation?. J. Epidemiol. Commun. Health.

[94] WHO (World Health Organization) Noncommunicable Diseases. http://www.searo.who.int/bangladesh/noncommunicable-diseases/en/.

[95] Chowdhury M.A.B., Uddin M.J., Haque M.R., Ibrahimou B. (2016). Hypertension among adults in Bangladesh: Evidence from a national cross-sectional survey. BMC Cardiovasc. Disord..

[96] Biswas T., Pervin S., Tanim M.I.A., Niessen L., Islam A. (2017). Bangladesh policy on prevention and control of non-communicable diseases: A policy analysis. BMC Public Health.

[97] Zaman M.M., Rahman M.M., Rahman M.R., Bhuiyan M.R., Karim M.N., Chowdhury M.A.J. (2016). Prevalence of risk factors for non-communicable diseases in Bangladesh: Results from STEPS survey 2010. Indian J. Public Health.

[98] Diabetic Association of Bangladesh Diabetic Association of Bangladesh-Magazine Kanti. https://www.dab-bd.org/kanti.php.

[99] Mendenhall E., Kohrt B.A., Norris S.A., Ndetei D., Prabhakaran D. (2017). Non-communicable disease syndemics: Poverty, depression, and diabetes among low-income populations. Lancet.

[100] Pfeiffer B.A., Clay S.W., Conatser R.R. (2001). A Green Prescription Study. J. Aging Health.

[101] Browning M., Lee K. (2017). Within what distance does “greenness” best predict physical health? A systematic review of articles with gis buffer analyses across the lifespan. Int. J. Environ. Res. Public Health.

[102] Ripley B., Bill V., Douglas M., Bates K.H., Albrecht G., David Firth M.B.R. (2013). Package “MASS”. ftp://192.218.129.11/pub/CRAN/web/packages/MASS/MASS.

[103] Fox J., Weisberg S., Adler D., Bates D., Baud-Bovy G., Ellison S., Firth D., Friendly M., Gorjanc G., Graves S. (2016). Package ‘Car’. https://cran.microsoft.com/snapshot/2017-06-17/web/packages/car/car.

[104] Tingley D., Yamamoto T., Hirose K., Keele L., Imai K. (2014). Mediation: R package for causal mediation analysis. J. Stat. Softw..

[105] Dzhambov A.M., Browning M.H., Markevych I., Hartig T., Lercher P. (2020). Analytical approaches to testing pathways linking greenspace to health: A scoping review of the empirical literature. Environ. Res..

[106] Imai K., Keele L., Tingley D. (2010). A General Approach to Causal Mediation Analysis. Psychol. Methods.

[107] Klompmaker J.O., Janssen N.A.H., Bloemsma L.D., Gehring U., Wijga A.H., Brink C., Van den Lebret E., Brunekreef B., Hoek G. (2019). Associations of combined exposures to surrounding green, air pollution, and road traffic noise with cardiometabolic diseases. Environ. Health Perspect..

[108] Quddus M., Rahman F., Monsuur F., de Ona J., Enoch M. (2019). Analyzing Bus Passengers’ Satisfaction in Dhaka using Discrete Choice Models. Transp. Res. Rec. J. Transp. Res. Board.

[109] Labib S.M., Mohiuddin H., Shakil S.H. (2013). Transport Sustainability of Dhaka: A Measure of Ecological Footprint and Means for Sustainable Transportation System. J. Bangladesh Inst. Planners.

[110] Klompmaker J.O., Hoek G., Bloemsma L.D., Gehring U., Strak M., Wijga A.H., van den Brink C., Brunekreef B., Lebret E., Janssen N.A.H. (2018). Green space definition affects associations of green space with overweight and physical activity. Environ. Res..

[111] Pietilä M., Neuvonen M., Borodulin K., Korpela K., Sievänen T., Tyrväinen L. (2015). Relationships between exposure to urban green spaces, physical activity and self-rated health. J. Outdoor Recreat. Tour..

[112] Shuvo F.K., Feng X., Akaraci S., Astell-Burt T. (2020). Urban green space and health in low and middle-income countries: A critical review. Urban For. Urban Green..

[113] Jansen M., Kamphuis C.B.M., Pierik F.H., Ettema D.F., Dijst M.J. (2018). Neighborhood-based PA and its environmental correlates: A GIS- and GPS based cross-sectional study in the Netherlands. BMC Public Health.

[114] Broyles S.T., Mowen A.J., Theall K.P., Gustat J., Rung A.L. (2011). Integrating social capital into a park-use and active-living framework. Am. J. Prev. Med..

[115] Hong A., Sallis J.F., King A.C., Conway T.L., Saelens B., Cain K.L., Fox E.H., Frank L.D. (2018). Linking green space to neighborhood social capital in older adults: The role of perceived safety. Soc. Sci. Med..

[116] Jennings V., Bamkole O. (2019). The relationship between social cohesion and urban green space: An avenue for health promotion. Int. J. Environ. Res. Public Health.

[117] Reubi D., Herrick C., Brown T. (2016). The politics of non-communicable diseases in the global South. Heal. Place.

[118] Milà C., Ranzani O., Sanchez M., Ambrós A., Bhogadi S., Kinra S., Kogevinas M., Dadvand P., Tonne C. (2020). Land-Use Change and Cardiometabolic Risk Factors in an Urbanizing Area of South India: A Population-Based Cohort Study. Environ. Health Perspect..

[119] Nieuwenhuijsen M.J., Khreis H., Triguero-Mas M., Gascon M., Dadvand P. (2017). Fifty shades of green. Epidemiology.

[120] Shuvo F.K., Hakim S.S. (2013). A Proposed Framework for Regenerating Urban Green in Dhaka City. J. Bangladesh Inst. Planners.

[121] Sultana R., Birtchnell T., Gill N. (2020). Urban greening and mobility justice in Dhaka’s informal settlements. Mobilities.

[122] Nahar K. (2019). Park-Playground Project: DSCC Revises Up Cost, Extends Tenure. The Financial Express.

[123] The World Bank Bangladesh-Dhaka City Neighborhood Upgrading Project: A More Liveable Dhaka City (English) The World Bank. http://documents.worldbank.org/curated/en/382111568359904697/Bangladesh-Dhaka-City-Neighborhood-Upgrading-Project-A-More-Liveable-Dhaka-City.

[124] Rigolon A., Browning M., Jennings V. (2018). Inequities in the quality of urban park systems: An environmental justice investigation of cities in the United States. Landsc. Urban Plan..

[125] Wolch J.R., Byrne J., Newell J.P. (2014). Urban green space, public health, and environmental justice: The challenge of making cities “just green enough”. Landsc. Urban Plan..

[126] Rigolon A., Németh J. (2018). “We’re not in the business of housing:” Environmental gentrification and the nonprofitization of green infrastructure projects. Cities.

[127] Hartig T., Mitchell R., de Vries S., Frumkin H. (2014). Nature and Health. Annu. Rev. Public Health.

[128] Becker D.A., Browning M.H.E.M., Kuo M., Van Den Eeden S.K. (2019). Is green land cover associated with less health care spending? Promising findings from county-level Medicare spending in the continental United States. Urban For. Urban Green..

[129] Buckley R., Brough P., Hague L., Chauvenet A., Fleming C., Roche E., Sofija E., Harris N. (2019). Economic value of protected areas via visitor mental health. Nat. Commun..

[130] Chen X. (2020). Monetary valuation of urban nature’s health effects: A systematic review. J. Environ. Plan. Manag..

